# ALAIN01—Alemtuzumab in autoimmune inflammatory neurodegeneration: mechanisms of action and neuroprotective potential

**DOI:** 10.1186/s12883-016-0556-9

**Published:** 2016-03-10

**Authors:** Tobias Ruck, Ali Maisam Afzali, Karl-Friedrich Lukat, Maria Eveslage, Catharina C. Gross, Steffen Pfeuffer, Stefan Bittner, Luisa Klotz, Nico Melzer, Heinz Wiendl, Sven G. Meuth

**Affiliations:** Department of Neurology, University of Münster, Albert-Schweitzer-Campus 1, 48149 Münster, Germany; Center for Clinical Studies, University of Münster, Münster, Germany; Institute of Biostatistics and Clinical Research, University of Münster, Münster, Germany

**Keywords:** Alemtuzumab, Multiple sclerosis, Disease-modifying therapy, Risk stratification, Mechanism of action, Secondary autoimmune disease

## Abstract

**Background:**

Alemtuzumab (Lemtrada®) is a newly approved therapeutic agent for relapsing-remitting multiple sclerosis (RRMS). In previous phase II and III clinical trials, alemtuzumab has proven superior efficacy to subcutaneous interferon beta-1a concerning relapse rate and disability progression with unprecedented durability and long-lasting freedom of disease activity. The humanized monoclonal antibody targets CD52, leading to a rapid and long-lasting depletion, especially of B and T cells. Arising from hematopoietic precursor cells a fundamental reprogramming of the immune system restores tolerogenic networks effectively suppressing autoimmune inflammatory responses in the central nervous system (CNS). Despite its favourable effects alemtuzumab holds a severe risk of side effects with secondary autoimmunity being the most considerable. Markers for risk stratification and treatment response improving patient selection and therapy guidance are a big unmet need for MS patients and health care providers.

**Methods/design:**

This is a mono center, single arm, explorative phase IV study including 15 patients with highly active RRMS designed for 3 years. Patients will be studied by a high-resolution analysis comprising a repertoire of various immunological assays for the detection of immune cells and their function in peripheral blood as well as the cerebrospinal fluid (CSF). These assays encompass a number of experiments investigating immune cell subset composition, activation status, cytokine secretion, migratory capacity, potential neuroprotective properties and cytolytic activity complemented by instrument-based diagnostics like MRI scans, evoked potentials and optical coherence tomography (OCT).

**Discussion:**

Our study represents the first in-depth and longitudinal functional analysis of key immunological parameters in the periphery and the CNS compartment underlying the fundamental effects of alemtuzumab in MS patients. By combining clinical, experimental and MRI data our study will provide a deeper understanding of alemtuzumab’s mechanisms of action (MOA) potentially identifying immune signatures associated with treatment response or the development of secondary autoimmunity. After validation in larger cohorts this might help to improve efficacy and safety of alemtuzumab therapy in RRMS patients.

**Trial registration:**

NCT02419378 (clinicaltrials.gov), registered 31 March 2015.

**Electronic supplementary material:**

The online version of this article (doi:10.1186/s12883-016-0556-9) contains supplementary material, which is available to authorized users.

## Background

Approximately 2.5 million people around the world suffer from multiple sclerosis (MS) [1], a chronic inflammatory disorder of the central nervous system (CNS). Inflammation, demyelination, and axonal degeneration are distinctive features of the pathological mechanism [2]. Its clinical course is typically characterized by initial episodes of transient neurological deficits with full or partial recovery (relapsing-remitting MS); over the time deficits may cumulate to increasing disability. With the development of new therapeutic agents treatment goals are changing from solely stabilizing to reversing the disease.

Alemtuzumab is a new promising therapy for RRMS, which might be able to fulfil this aim. Alemtuzumab has been approved by the EMA (European Medicines Agency) in September 2013 and the FDA (U.S. Food and Drug Administration) in November 2014 for the treatment of RRMS under the name Lemtrada®. It is a humanized monoclonal antibody targeting CD52 primarily expressed on T- and B-lymphocytes, and to lesser extents on dendritic cells, monocytes and macrophages [3]. Alemtuzumab leads to a rapid and significant depletion of CD52 expressing cells by antibody-dependent cell-mediated and complement-dependent cytotoxicity [4, 5]; hematopoietic precursor cells are not affected. Subsequent to depletion a slow repopulation starts arising from hematopoietic precursor cells following a distinct pattern. B cells repopulate rapidly within 6 months with predominating immature B cells, prolonged CD27^+^ memory B cell lymphopenia and increased serum levels of BAFF (B cell activating factor) [6]. In contrast, CD4^+^ and CD8^+^ lymphocytes do not recover to the lower limit of normal (LLN) before 35 or 20 months respectively post treatment [7]. CD4^+^ memory cells predominate in the CD4^+^ cell population probably based on homeostatic proliferation of cells that escaped depletion. Similarly, CD4^+^CD25^high^CD127^dim/-^ regulatory T cells (Treg) are found to be relatively upregulated 9 months after alemtuzumab treatment [7, 8]. In contrast, the proportion of proinflammatory cell-types such as T helper (T_h_) 1 and T_h_ 17 cells and the serum levels of their respective cytokines are reduced [9]. The reduced levels of circulating B and T cells after alemtuzumab treatment accompanied by successive repopulation restoring immune tolerance networks are assumed to underlie its strong anti-inflammatory effects in multiple sclerosis.

Several previous studies have proven high efficacy of alemtuzumab in early stages of multiple sclerosis before the course of pathophysiological events causes permanent CNS damage. In initial studies with secondary progressive MS (SPMS) patients alemtuzumab was not able to slow disease progression indicating a clear window of therapeutic opportunity in early disease stages [10–13]. Therefore, the following clinical development program included only RRMS patients.

The Phase 2, randomized, comparator-controlled study of treatment-naïve patients with early, active RRMS, CAMMS223 showed that patients treated with alemtuzumab (12 and 24 mg dose groups pooled) experienced a 74 % reduction in the risk for relapse (*p* < 0.001) and a 71 % reduction in the risk for sustained accumulation of disability (SAD; defined as an increase of 1.5 points for patients with a baseline score of 0 and of at least 1.0 point for patients with a baseline score of 1.0 or more for at least 6 months) (*p* < 0.001) compared to patients treated with subcutaneous (sc) IFNb-1a. Alemtuzumab patients also experienced a significant reduction in lesion burden (T2 weighted magnetic resonance imaging [MRI] findings) compared with sc IFNb-1a patients (*p* = 0.005). Moreover, EDSS (Expanded Disability Status Scale) scores improved by 0.39 points in alemtuzumab patients but worsened by 0.38 points in the IFNb-1a patients (*p* < 0.001) [14]. A post-hoc analysis demonstrated that even patients without clinical activity of MS before or during the trial showed improved EDSS scores and prompted hypotheses on an active neuroprotective potential of alemtuzumab [15].

The two phase 3 trials CARE-MS I [16] and CARE-MS II [17] again compared alemtuzumab with IFNb-1a therapy sc and included treatment-naive or pre-treated RRMS patients. Most of the results of CAMMS223 were confirmed. Alemtuzumab demonstrated a superior reduction of clinical relapse activity, with a risk reduction of 54.9 % in CARE-MS I and 49.4 % in CARE-MS II. However, only in CARE-MS II (42 % decreased risk; 21.1 % with IFNb-1a versus 12.7 % with alemtuzumab) but not in CARE-MS I (11 % with IFNb-1a versus 8 % with alemtuzumab) a significant reduction in the risk for SAD was observed.

Meanwhile, long-term data from on-going extension studies of patients from Phase II and III trials have been published confirming previous observations. The 5-year follow-up of CAMMS223 showed sustained beneficial effects on relapse rate and disability without almost any MRI alterations. Reduction in annual relapse reduction (ARR) by 69 % (*p* < 0.0001) was observed as well as reduction in SAD by 72 % (*p* < 0.0001) with stable or improved EDSS scores in 74 % of patients [17]. Recent 4-year follow-up data from CARE-MS extension studies demonstrated that 50–60 % of patients showed no evidence of disease activity (NEDA), which was defined as absence of clinical disease and MRI activity, in years 3 and 4. After the initial two treatment courses 36 % of patients in CARE-MS I and 32 % of patients in CARE-MS II required retreatment over 3 years, whereas 2–5 % of patients received other disease-modifying therapies (DMT) [18, 19]. Furthermore, the 4-year follow-up of CARE-MS II demonstrated favourable effects on disability with 6-month sustained reduction in disability (SRD, defined as a reduction in the EDSS score of either ≥1.0 or 0.5, for baseline EDSS scores below and above 5.5 for at least 6 months) in 41 % of patients and 12-month SRD in 30 % over 4 years [20]. Around 70 % of patients were free of any MRI disease activity at years 3 and 4 [21, 22]. Patients switched from IFNb-1a to alemtuzumab, experienced substantial improvements in clinical as well as MRI parameters [23–27].

However, the advantages of alemtuzumab are opposed by considerable side effects. Alemtuzumab was associated with secondary autoimmunity in all mentioned and previous studies. Thyroid adverse events were most common (18 % in CARE-MS I, 17 % in CARE-MS II, 26 % in CAMMS223, 41 % in the 7-year follow-up of the Cambridge-group) [16, 17, 28]. Immune thrombocytopenic purpura (ITP) (1–3 %) and anti-glomerular basement membrane disease (<1 %, anti-GBM, also known as Goodpasture’s syndrome) were also observed. More frequently, higher rates of infections (66 %), especially mild to moderate upper and lower airway infections, and infusion-associated reactions (IAR) (90 %) occurred in alemtuzumab treated patients [14, 16, 17]. The experiences of these trials led to the implementation of an extensive surveillance program allowing for early recognition and therapy of adverse events.

Although alemtuzumab is a promising and valuable new therapy for RRMS, its long-lasting potentially irreversible effect and the risk of severe side effects demand reliable markers for risk stratification and treatment response improving patient selection and therapy guidance. Thus, it is indispensable to shed more light on the still not fully understood MOA [29].

## Methods/design

### Study design

ALAIN01 is a single center, single arm, explorative phase IV study to elucidate further insights into the MOA of alemtuzumab treatment in 15 patients with RRMS. The study center is the Department of Neurology at the University Hospital Münster, Germany. Recruitment started in May 2015.

After a screening period of at most 28 days, eligible patients will start investigational treatment. The study is designed for 3 years and efficacy and safety measures are assessed on at least half-yearly visits. Additionally, blood and urine samples will be collected monthly to monitor for autoimmune diseases, which may be carried out by a local physician.

As required by the summary of product characteristics (SmPC) for Lemtrada®, patients will be advised to continue monitoring for late undesirable effects of Lemtrada® after termination of study participation. The study site will provide post-study patient care but also support continuation of medical attendance by a resident neurologist of the patient’s choice.

The clinical study is registered at clinicaltrials.gov (NCT02419378). It will be performed in accordance with the approval of the local ethics committee (Ethik-Kommission der Ärztekammer Westfalen-Lippe und der Westfälischen Wilhelms-Universität Münster, 2014-545-f-A), the requirements of the current German drug law (“Arzneimittelgesetz”), the current legal provisions regarding data protection, and the principals of Good Clinical Practice.

All participants are obliged to hand in the signed informed consent form (ICF).

### Patient population and selection

Patients with highly active RRMS will be included in the study. The below eligibility criteria imply, that they are indicated to receive alemtuzumab irrespective of study participation.

Patients who do not meet all the inclusion criteria in Table 1 or meet one of the exclusion criteria mentioned in Table 2 will be excluded from further study participation. Patients will also be excluded if treatment with alemtuzumab has not been started within 28 days after signing the informed consent form. Reasons for exclusion will be recorded in the CRF. Excluded patients may be re-screened at a later time.

**Table 1:** Inclusion Criteria

Patients must meet all of the following criteria to be eligible for further study participation:	
1. Signed informed consent form (ICF)	
2. Age 18 to 55 years old (inclusive) as of the date the ICF is signed	
3. Diagnosis of MS according to the McDonald criteria 2010 and cranial MRI scan demonstrating white matter lesions attributable to MS within 10 years before screening	
4. Onset of MS symptoms (as determined by a neurologist, either at present or retrospectively) within 10 years of the date the ICF is signed	
5. EDSS score 0.0 to 5.0 (inclusive) at Screening	
6. Patients with (highly) active RRMS disease course indicated to receive alemtuzumab according to the following conditions (at least 1 out of 3 conditions has to be fulfilled):	
1. ≥2 MS relapses within 24 months	
2. Clinical (≥1 relapse) or MRI (new gadolinium enhancing lesions) disease activity under therapy with other disease-modifying therapies	
3. Severe relapse with high disease activity (≥9 T2 hyperintense Lesions and ≥1 gadolinium enhancing lesion) on MRI.	
7. Completion of all vaccinations required by the applicable immunization guidelines published by “ständige Impfkommission” (STIKO)	
8. History of chickenpox or positive test for antibodies against varizella zoster virus (VZV)

**Table 2:** Exclusion Criteria

Patients will be excluded from this study if they meet any of the following exclusion criteria:	
1. Participation in another clinical trial at present or within 4 weeks of study entry. There may be exceptions at the discretion of the Investigator.	
2. Has any progressive form of MS	
3. Hypersensitivity to the active substance, or to any of the excipients of Lemtrada®	
4. Medical, psychiatric, cognitive, or other conditions that, in the Investigator’s opinion, compromise the patient's ability to understand the patient information, to give informed consent, to comply with the trial protocol, or to complete the study	
5. Any disability acquired from trauma or another illness that could interfere with evaluation of disability due to MS	
6. Major systemic disease or other illness that would, in the opinion of the Investigator, compromise patient safety or interfere with the interpretation of study results, e.g., current peptic ulcer disease or other conditions that may predispose to haemorrhage	
7. Known bleeding disorder (e.g., dysfibrinogenemia, factor IX deficiency, haemophilia, Von Willebrand’s disease, disseminated intravascular coagulation (DIC), fibrinogen deficiency, or clotting factor deficiency)	
8. Significant autoimmune disease including but not limited to immune cytopenias, rheumatoid arthritis, systemic lupus erythematosus, other connective tissue disorders, vasculitis, inflammatory bowel disease, severe psoriasis	
9. History of malignancy, except basal skin cell carcinoma	
10. Major psychiatric disorder that is not adequately controlled by treatment	
11. Epileptic seizures that are not adequately controlled by treatment	
12. Active infection, e.g., deep-tissue infection, that the Investigator considers sufficiently serious to preclude study participation	
13. In the Investigator’s opinion, is at high risk for infection (e.g., indwelling catheter, dysphagia with aspiration, decubitus ulcer, history of prior aspiration pneumonia or recurrent urinary tract infection)	
14. Seropositivity for human immunodeficiency virus (HIV)	
15. Infection with hepatitis C virus	
16. Past or present hepatitis B infection (positive hepatitis B serology)	
17. Active infection with human cytomegaly virus (HCMV), Epstein-Barr virus (EBV), varicella-zoster virus (VZV)	
18. Latent tuberculosis unless effective anti-tuberculosis therapy has been completed, or active tuberculosis.	
19. Invasive fungal infections in history and at present	
20. Cervical cytology other than PAP I or PAP II (Papanicolaou) or cervical high risk human papillomavirus (HPV) positivity	
21. Any other illness or infection (latent or active) that, in the Investigator’s opinion, could be exacerbated by study medication	
22. CD4^+^ T-cell count (absolute CD3^+^CD4^+^) < lower limit of normal (LLN) at Screening	
23. CD8^+^ T-cell count (absolute CD3^+^CD8^+^) < LLN at Screening	
24. B-cell count (absolute CD19^+^) < LLN at Screening	
25. Absolute neutrophil count < LLN at Screening	
26. Confirmed platelet count < the LLN of the evaluating laboratory at Screening or documented at <100,000/μL within the past year on a sample without platelet clumping	
27. Presence (i.e., above the ULN) of anti-thyroid stimulating hormone receptor antibodies (anti-TSHR) and anti-thyroid peroxidase antibody (anti-TPO)	
28. Any hepatic or renal function value grade 2 or higher at Screening, with the exception of hyperbilirubinemia due to Gilbert’s syndrome. See Table below, drawn from the National Cancer Institute (NCI) Common Terminology Criteria for Adverse Events v4.0 (CTCAE), published 28 May 2009.	
29. Vaccination less than 6 weeks prior to treatment with Lemtrada®.	
30. Treatment with antineoplastic or immunosuppressive drugs within 8 weeks prior to study inclusion	
31. Intolerance of pulsed corticosteroids, especially a history of steroid psychosis	
32. Inability to undergo MRI with gadolinium administration	
33. Of childbearing potential with a positive serum pregnancy test, pregnant or lactating	
34. Female patients of childbearing potential: Unwilling to agree to use a reliable and acceptable contraceptive method (Pearl index <1) throughout the study period. These methods include: hormone releasing intrauterine device (IUD), hormonal-based contraception, surgical sterilization, abstinence, or double-barrier contraception (condom and occlusive cap [diaphragm or cervical cap combined with spermicide]).	

### Interventions

After a screening period of approximately 4 weeks for determination of patient’s eligibility, included patients will be participating in the study for 36 months with alemtuzumab infusions at month 0 and 12.

Apart from clinical examination screening comprises visual, somatosensory and motor evoked potentials, optical coherence tomography, MRI scans, blood and cerebrospinal fluid (CSF) sampling. Female patients of child bearing potential are obliged to have a pregnancy test prior to each course as well as yearly endometrial smear tests and investigations for human papillomavirus (HPV). All follow-up examinations will be performed as scheduled according to the Additional file 1: Assessment Schedule.

Patients will be treated with Lemtrada® by daily IV infusions over a period of approximately 4 h in a supervised medical setting. It will be administered at a dosage of 12 mg/day for 5 consecutive days at Month 0 (60 mg total dose) and for 3 consecutive days at Month 12 (36 mg total dose). Observation for infusion-associated reactions (IARs) is recommended during and for 2 h after Lemtrada® infusion.

As an IAR precaution patients will be pre-treated with corticosteroids (1000 mg prednisolone) immediately prior to administration of Lemtrada® on each of the first 3 days of each treatment course. Additionally, pre-treatment with antihistamines and/or antipyretics prior to administration of Lemtrada® may be considered. Oral prophylaxis for herpes infection will be administered to all patients starting on the first day of each treatment course and continuing for a minimum of 1 month following treatment with Lemtrada®.

### Outcome parameters

#### Primary endpoints

Absolute and relative change from baseline of T cell subsets (CD4 and CD8 positive T cells: naïve T cells, T effector cells, T memory cells, regulatory T cells; T-helper subsets: T_h_ 1, T_h_ 2, T_h_ 17), B cell subsets (Recent bone marrow emigrants, mature naïve, memory B cells and plasma cells), natural killer cells (CD56bright, CD56dim, natural killer T cells), antigen-presenting cells (Dendritic cells: CD303^+^ plasmacytoid, CD11c^+^ and CD141^+^ myeloid dendritic cells; non-classical CD14^+^CD16^++^HLA-DR^++^, classical CD14^++^CD16^−^HLA-DR^+^ monocytes and macrophages) and myeloid-derived suppressor cells in the peripheral blood at indicated time-points of sampling are considered as primary endpoints (see Additional file 1: Assessment Schedule). A complete listing is found in Table 3.

**Table 3:** Primary Endpoints

Absolute and relative change from baseline of the following cell counts in the peripheral blood at indicated time points of sampling (see Additional file 1: Assessment Schedule):	
a. T cell subsets:	
- CD4 and CD8 positive T cells: naïve T cells, T effector cells, T memory cells, regulatory T cells	
- T-helper subsets: T_h_ 1, T_h_ 2, T_h_ 17	
b. B cell subsets:	
- Recent bone marrow emigrants, mature naïve, memory B cells	
- Plasma cells	
c. Natural killer cells:	
- CD56bright, CD56dim	
- Natural killer T cells	
d. Antigen-Presenting cells:	
- Dendritic cells: CD303^+^ plasmacytoid, CD11c^+^ and CD141^+^ myeloid dendritic cells	
- Monocytes and macrophages (non-classical CD14^+^CD16^++^HLA-DR^++^, classical CD14^++^CD16^−^HLA-DR^+^)	
e. Myeloid-derived suppressor cells.	

#### Secondary endpoints

Secondary endpoints extend the already mentioned investigations in the peripheral blood and mainly focus on three points: The absolute and relative change from baseline of the aforementioned cell counts in the CSF at indicated time points of sampling (see Additional file 1: Assessment Schedule), functional characterization of T cells in peripheral blood and the CSF at indicated time points of sampling (see Additional file 1: Assessment Schedule) and the evaluation of potential neuroprotective properties of alemtuzumab. A complete listing is found in Table 4.

**Table 4:** Secondary Endpoints

1. Absolute and relative change from baseline of cell-counts in the CSF at indicated time-points of sampling (see Additional file 1: Assessment Schedule). The same cell-types as indicated for the primary endpoints will be evaluated.	
2. Functional characterization of T-cells in the peripheral blood and the CSF at indicated time-points of sampling (see Additional file 1: Assessment Schedule):	
a. Activation status of cell surface receptors assessed by flow cytometry: Relative and absolute change from baseline of mean fluorescence intensity (MFI) and of proportion of positive cells regarding CD25, HLA-DR, LFA-1, CD29, CD69, CD71 expression	
b. Expression of co-inhibitory molecules assessed by flow cytometry: Relative and absolute change from baseline of MFI and of proportion of positive cells regarding PD-1 = CD279, ICOS = CD278, TIM-3, CTLA4 expression	
c. Effector functions of CD4 and CD8 positive T cells:	
- Relative and absolute change from baseline of the results of cell proliferation assays assessed as percentage of proliferated cells	
- Relative and absolute change from baseline of cytokine production measurement assessed as concentration	
- Relative and absolute change from baseline of cytolytic activity assessed by flow cytometry measurement of MFI and proportion of positive cells regarding Granzyme B, Perforin and CD107a expression	
- Relative and absolute change from baseline of intracellular calcium response assessed as concentration	
d. Migrational capacity:	
- Relative and absolute change from baseline MFI and proportion of positive cells assessed by flow cytometry expression analysis of CD11a, CD31, CD44, CD49d, CCR5, CCR6, CCR7	
- Absolute and relative change of cell numbers of migrated cells compared to baseline assessed in an in vitro model by flow cytometry analysis	
e. Spectratyping of the T cell repertoire concerning the expansion of distinct clones:	
- Relative and absolute change from baseline for complexity scores	
- Qualitative comparison of the distribution of CDR3 sequences	
f. Regulatory T-cell function:	
- Relative and absolute change from baseline in production of TGF-beta and IL-10 of CD4^+^CD25^+^FOXP3^+^ regulatory T cells	
- Suppression of T cell proliferation: Relative and absolute change from baseline in responder T cell proliferation assessed by suppression assays	

##### Cell Isolation

PBMCs (peripheral blood mononuclear cells) will be isolated from peripheral venous blood (90 ml EDTA blood) by centrifugation on a Lymphoprep™ (Fresenius Kabi Norge AS) density gradient. Purified PBMC will be aliquoted and cryo-preserved in liquid nitrogen for at least 4 weeks before analysis (except for the assessment of immune cell composition, where freshly isolated cells will be used).

CSF will be obtained from patients by lumbar puncture and centrifuged within 30 min. The supernatant will be separated and cells will be used for further analyses.

##### Flow cytometry

The identification and quantification of leukocyte subsets will be done by flow cytometric analysis of PBMCs and CSF cells as described [30]. For intracellular stainings, the BD Cytofix/Cytoperm™ Fixation/Permeabilization Solution Kit with BD GolgiPlug™ will be used (BD Biosciences) according to standard protocols.

Cells will be analysed on a Gallios Flow Cytometer (Beckman Coulter).

##### CDR3 spectratyping

Spectratyping will be performed essentially as described before [31] using a common Vβ nomenclature [32]. Spectratyping data will be processed by GeneMarker Software V1.91 (SoftGenetics). For all PCR products of Vβ- and Cβ-primers the peak intensities will be plotted against the fragment lengths. Unskewed repertoires yield Gaussian length distributions with a high number of detectable peaks, while skewed repertoires show distortions with reduced numbers of fragment lengths. A previous study [33] using capillary electrophoresis revealed maximum peak numbers between 9 and 13 for each individual of the 24 inspected Vβs giving a total of 287 peaks, representing a theoretical complexity of 100 %. The complexity score (CS) will be calculated by quantifying the obtained peaks in every single Vβ of each sample and dividing the peak number through the theoretical maximum.

##### Migration Assay

Transmigration assays will be performed with minor modifications as described previously [34, 35]. Briefly, transwell inserts will be assembled with HBMEC (human brain microvascular endothelial cells). Human PBMCs (each 5x105) will be transferred to the HBMEC layer for 14 h. Migrated cells from the lower of the two compartments will be collected and Calibrite beads (BD Biosciences) will be added. Next, human cells will be stained for CD4, CD8, CD14, CD19 and CD56, with the respective monoclonal antibodies and relative cell numbers will be determined by flow cytometry.

##### Proliferation Assays

Human PBMCs will be labelled with 5 μM carboxyfluorescein succinimidyl ester (CFSE; Invitrogen) according to the manufacturer’s instructions, and proliferation will be assessed by flow cytometry after stimulation with anti-human CD3 (OKT3; 2 μg/ml) and soluble mouse anti-human CD28 (1 μg/ml; eBioscience) for 4 days.

Alternatively cell proliferation will be assessed using the ATPLite™ Luminescence Assay System (PerkinElmer) according to the manufacturer’s instructions. Luminescence will be measured on a TopCount NXT.

##### Intracellular calcium imaging

For intracellular calcium imaging experiments lymphocytes will be isolated using magnetic-activated cell sorting. All measurements will be performed in HEPES buffer containing (in mM): NaCl, 120; KCl, 2.5; NaH2PO4, 1.25; HEPES, 30; MgSO4, 2; glucose, 10; pH 7.25 and osmolarity will be set to 305 mOsm/kg. Cells will be loaded with 5 μM Fura-2 AM (Invitrogen) for 30 min at 37 °C. Anti-mouse CD3 (clone 145-2C11; eBioscience, 10 μg/ml) will be added after 15 min and fluorescence will be measured with a TECAN infinite M200Pro fluorimeter (Tecan Group Ltd.). Excitation will be alternated between 340 and 380 nm and emission will be measured at 509 nm.

##### Cytokine detection

Appropriate ELISA-Kits (enzyme linked immunosorbent assay) will be used to detect cytokine concentrations in cell culture supernatants and cerebrospinal fluid. Alternatively fluorescent bead immunoassays (FlowCytomix, eBioscience) will be used to quantify the production of the chemokines and cytokines according to the manufacturer’s instructions.

##### Suppression Assay

CD4^+^CD25^+^CD127^dim/-^ regulatory T cells will be separated from responder T cells by magnetic-activated cell sorting using a CD4^+^CD25^+^CD127^dim/-^ Regulatory T Cell Isolation Kit II (Miltenyi Biotec). Responder T cells will be labelled with 5 μM carboxyfluorescein succinimidyl ester (CFSE; Invitrogen) according to the manufacturer’s instructions. Afterwards regulatory T cells and CFSE^+^ responder T cells will be cocultured at a ratio of 1:2 and 1:1. Cultures will be stimulated with anti-human CD3 (OKT3; 2 μg/ml) and soluble mouse anti-human CD28 (1 μg/ml; eBioscience) for 4 days. Proliferation will be assessed by flow cytometry after stimulation.

##### Concentration of Neurotrophic Factors

Investigation of the concentration of the following neurotrophic factors in peripheral blood and cerebrospinal fluid: nerve growth factor (NGF), brain-derived neurotrophic factor (BDNF), neurotrophin-3 (NT-3), and neurotrophin-4 (NT-4), ciliary neurotrophic factor (CNTF).

Cell culture supernatants and CSF will be interrogated for neurotrophins by appropriate ELISA kits according to manufacturer’s instructions.

##### Quantification of markers of neurodegeneration

Appropriate ELISA kits will be used to detect S100β, Tau, phospho-Tau, β-Amyloid, Neurofilament (low weight), Neurofilament (high weight), N-acetylaspartate (NAA), Tubulin, Actin, neuron-specific enolase (NSE) and glial fibrillary acidic protein (GFAP).

##### Multi-electrode Array

To analyse the impact of cell culture supernatants or cerebrospinal fluid on activity of neuronal networks, different parameters of neuronal network activity will be determined using the multi-electrode array technique [36–38]. Multi-electrode array detects compound field potentials in the spatial vicinity of multiple extracellular electrodes in densely cultured hippocampal neurons, which are capable of generating spontaneous synchronized activity. A complete listing is found in Table 5.

**Table 5:** Additional Endpoints

The following additional endpoints will undergo exploratory evaluation:	
1. Number of patients who experienced sustained accumulation of disability (SAD) during the study period (Study period: From the day of treatment initiation until month 36 visit. SAD: for patients with a Baseline EDSS score of 0.0, SAD is defined as an increase of ≥1.5 points sustained over a 6-month consecutive period. For patients with a Baseline EDSS score of ≥1.0, SAD is defined as an increase of ≥1.0 point sustained over a 6-month consecutive period.)	
2. Time to SAD	
3. Time to sustained reduction in disability (SRD) based on EDSS scores (SRD: a ≥1 point decrease on the EDSS sustained for 6 consecutive months for patients with a baseline EDSS ≥2)	
4. Absolute Number of relapses during the study period	
5. Number or relapses which occurred during the study period and required corticosteroid therapy	
6. Proportion of patients who are relapse free at year 3	
7. Time to first relapse	
8. Absolute and relative change from baseline in the following functional scores at each time point of assessment after treatment initiation (see Additional file 1: Assessment Schedule): EDSS, visual functional system score as part of the assessments for EDSS, MSFC and each MSFC component, FSMC and each subscore (mental and physical fatigue)	
9. The proportion of patients who have worsened, remained stable, or improved as indicated by change from Baseline in EDSS scores at the end of the study	
10. Change from baseline of the following HRQoL measures at each time point of assessment after treatment initiation (see Additional file 1: Assessment Schedule): FAMS, EQ-5D (total score, Physical Component summary Measure, Mental Component Summary Measure), SF-36	
11. The proportion of patients who have worsened, remained stable, or improved as indicated by change from Baseline in MSFC scores at the end of the study	
12. Absolute and relative change from baseline amplitudes [V] and latencies [s] of evoked potentials (VEP, SEP, MEP)	
13. Percent change from baseline in magnetic resonance imaging (MRI)-T2 hyperintense lesion volume at each time point of assessment after treatment initiation (see Additional file 1: Assessment Schedule)	
14. Number of new gadolinium-enhancing lesions on MRI-T1 in comparison to Baseline	
15. Number of new or enlarging hyperintense lesions measured by T2-weighted MRI in comparison to Baseline at each time point of assessment (see Additional file 1: Assessment Schedule)	
16. Absolute and relative change from baseline of IL-21 concentration in blood serum at indicated time-points of sampling	
17. Absolute and relative change from baseline of following parameters assessed in CSF: cell counts, concentration of lactate, protein, and immunoglobulines (IgA, IgG, IgM)	
18. Qualitative assessment of the change in presence of oligoclonal bands in CSF from baseline	
19. Proportion of patients with no MS disease activity (i.e., deterioration in MRI related endpoints, relapse) at the end of the study	

At our study center samples of highly controlled and validated groups of other MS therapy cohorts (e.g. natalizumab and fingolimod) and other neuroimmunological diseases (NMO, CIDP, autoimmune encephalitis) and healthy sex- and age-matched individuals are available as control groups. These control groups will be used for experiments, whenever it is possible and reasonable.

### Additional endpoints

Additional endpoints include clinical parameters, a number of extensive clinical scales as well as instrument-based diagnostics and are listed in detail in Table 5.

Patient disability will be evaluated at least at annual visits as specified in the Additional file 1: Assessment Schedule using EDSS as well as the specific criteria for SAD on the basis of the CAMMS223 study [14] accompanied by clinical outcome measurements applying the Multiple sclerosis functional composite (MSFC) and fatigue evaluation by the Fatigue Scale for Motor and Cognitive Functions (FSMC). The Health-Related Quality of Life (HRQoL) will be assessed by three self-report measures, the Functional Assessment of Multiple Sclerosis (FAMS), the EuroQol (EQ-5D) and the Short Form-36 (SF-36).

Instrument-based diagnostics encompass evoked potentials, optical coherence tomography and MRI scans including the rate of gadolinium-enhancing MRI lesions, total MRI-T2 lesion volume plus photon density and fluid attenuated inversion recovery (FLAIR) sequences as well as 3D-T1 and DTI acquisitions.

These investigations will be completed by measurements of serum concentrations of IL-21 at indicated time points (see Additional file 1: Assessment Schedule) by appropriate ELISA kits according to manufacturer’s instructions. Increased IL-21 concentrations in the serum have been associated with a higher risk for the development of secondary autoimmunity under alemtuzumab therapy. IL-21 is assumed to drive cycles of T cell expansion and apoptosis to excess leading to higher stochastic opportunities for T cells to encounter self-antigens, and hence autoimmune reactions. Therefore IL-21 might be used as a biomarker for the risk of developing autoimmunity providing essential information for clinical decisions [39].

### Sample size

Concerning the prevalence and the inclusion and exclusion criteria of this study, it seems feasible to recruit 15 patients over the course of 1 year. The absolute number of lymphocytes before and after treatment with alemtuzumab as described in previous studies [6, 8, 29] was used to assess the expected precision of the mean absolute number of cells estimation that can be attained with this number of patients:

The absolute number of lymphocyte cells before treatment is assumed to be 2100 ± 450 cells/μl (mean ± standard deviation). With a probability of 80 % a 95 % confidence interval for the mean number of lymphocyte cells measured in 15 patients before treatment would not exceed a range of ±284 cells/μl. With a probability of 80 % the 95 % confidence interval would not exceed a range of ±375 cells/μl in case samples of only 10 patients can be collected.

The absolute number of lymphocyte cells after treatment is expected to be lowered to 100 ± 50 cells/μl. With a probability of 80 % a 95 % confidence interval for the mean number of lymphocyte cells measured in 15 patients after treatment would not excess a length of ±32 cells/μl. With a probability of 80 % the 95 % confidence interval would not excess a length of ±42 cells/μl in case samples of only 10 patients can be collected.

The difference in absolute numbers of lymphocyte cells before and after treatment is assumed to be 2000 ± 500 cells/μl. With a probability of 80 % a 95 % confidence interval for the mean change in number of lymphocyte cells measured in 15 patients would not excess a length of ±315 cells/μl. With a probability of 80 % the 95 % confidence interval would not excess a length of ±417 cells/μl in case samples of only 10 patients can be collected.

The precision of this estimation is considered to be sufficient to track the condition of the immune system over time to describe the MOA of alemtuzumab treatment.

These calculations were performed using NQuery Advisor® 7.0.

### Statistical methods

Statistical analyses will be performed according to the principles of the ICH-guideline E9 “Statistical Principles for Clinical Trials” using standard statistical software like SAS or SPSS.

The statistical analysis will be performed according to the intention to treat principle (ITT analysis). In addition to the ITT-analysis a per protocol analysis (PP-analysis) will be performed. This analysis will include only those patients who could be treated with full adherence to the protocol.

The goal of this study is to detect the patterns in different populations of immune cells after treatment with alemtuzumab. Therefore, the analysis of the primary endpoints will focus on estimating the absolute and relative number of cells defined as primary endpoints using summary statistics such as mean and standard deviation, median and quartiles, or frequency and percent, as appropriate. The development over time will be displayed using boxplots. To assess the difference of absolute or relative number of cells between two different time points statistical tests appropriate to the statistical distribution of the particular endpoint will be used (Wilcoxon signed-rank test, Student’s *t*-test for paired samples, Sign-Test). In order to compare more than two successive measurements, the preferred methods of statistical analysis are Repeated Measures ANOVA, Mixed Models and Generalized Estimation Equations, appropriately accounting for intra-individual correlations.

Since this study is planned as an exploratory study, inferential statistics are intended to be exploratory (hypotheses generating), not confirmatory, and are interpreted accordingly. I.e., *p*-values are interpreted as a metric weight of evidence against the respective null hypothesis of no effect. Neither a global significance level nor local levels are determined. *P*-values are considered noticeable in case *p* < =0.05 and highly noticeable in case *p* < =0.01. These findings will be used to generate new hypotheses.

Statistical analyses of the pre-specified secondary endpoints will be performed with appropriate descriptive and inductive statistical methods using summary statistics such as mean and standard deviation, median and quartiles, or frequency and percent. Appropriate to the characteristics of the endpoints, statistical tests like Fisher's exact test, Chi-Square test, Mann—Whitney-*U* test, Wilcoxon signed-rank test or Student’s *t*-test will be performed.

Safety data will be evaluated descriptively, including all recruited study patients with at least one dose of the investigational product (safety population). Results will be reported by mean parameter estimates and associated 95 % confidence intervals. Adverse events of patients not receiving any investigational drug or adverse events occurring before first administration of investigational drug will be analysed separately.

## Discussion

Approved for the treatment of active RRMS alemtuzumab has demonstrated a favourable risk-benefit profile under the precautions of an intensive surveillance program. Alemtuzumab leads to a sustained reprogramming of the immune system lasting for at least several years and is associated with considerable adverse events, which necessitate the early recognition for therapy in time. For the best possible risk-benefit ratio deeper insights into the MOA are clearly needed to identify markers for treatment response and adverse event risk.

So far, the mechanism by which alemtuzumab exerts its therapeutic effects in MS is not fully elucidated. Initial clinical trials demonstrated efficiency of alemtuzumab rather in RRMS then SPMS pointing towards effects directed against acute inflammatory processes predominantly driven by adaptive immunity in contrast to neurodegenerative processes partially driven by innate immunity [40]. This is thought to be related to the limited expression of CD52 on innate immune cells leading to resistance to alemtuzumab mediated depletion. A study of Buggins and colleagues (2002) observed a loss of CD52 in tissue-resident human innate immune cells during maturation or differentiation [41]. The relatively unaffected innate immune system as well as the limited immune cell depletion in primary and secondary lymphoid tissues (as observed in humanized CD52 mice [4]) are believed to explain the low incidence of severe or opportunistic infections despite the long-lasting lymphopenia.

Besides quantitative effects, especially the qualitative effects of alemtuzumab reprogramming the immune system might underlie its long-lasting effects. CD4^+^CD25^high^CD127^low^ regulatory T cells have been demonstrated to be relatively increased in the CD4^+^ T cell compartment, although absolute numbers remain low hampering further investigations [7, 8]. Significantly increased levels of the immunoregulatory cytokines TGFβ-1 and IL-10 were observed. Furthermore, Th2 cells dominate the CD4^+^ T cell pool, while pro-inflammatory T_h_ 1 and T_h_ 17 cells are reduced in association with a reduction in IFN-γ, IL-12, IL-17, IL-21, IL-23 and IL-27 serum levels [42]. Jones and colleagues detected an increased expression of the inhibitory receptors PD-1 and LAG-3 on CD4^+^ T cells [7]. Our study intends to confirm and understand the underlying mechanisms of these findings as well as to expand them. Therefore a detailed immune phenotyping of different immune cell subsets and their properties is planned for the peripheral blood as well as the CSF. There has been no investigation of the CSF compartment so far under alemtuzumab treatment; however this promises deeper insight into the immune processes in the CNS.

The functional properties of T cells will be extensively investigated post alemtuzumab treatment including proliferation, cytokine production as well as immune regulatory and cytotoxic propensity. The spatial and functional changes observed over time will help to understand the reprogramming effects of alemtuzumab. The expression profiles of activating or inhibitory molecules on T cells will be assessed to gain insights into the fundamental alterations in immunological networks. The clonal expansion of autoreactive T cells is believed to be critically involved in the local immune reactions in MS [43]. A restoration of T cell diversity might therefore have beneficial effects to MS disease activity and will be assessed by spectratyping over study time.

Another focus of our study is on the migrational capacity of T cells after alemtuzumab treatment. It is assumed for MS that autoimmune T cells migrate into the CNS after they have been activated in the periphery [44–46]. A study of Havari and colleagues has recently shown that anti-murine CD52 treatment had no effect on the migratory ability of immune cells [47]. However, since results from animal experiments cannot be easily transferred these findings have to be further evaluated in humans. Moreover, CD52 has been shown to play a role in transendothelial migration of T cells through human umbilical vein endothelial cell monolayers [48].

More uncertainty surrounds the neuroprotective potential of alemtuzumab compared to anti-inflammatory effects. A post hoc subgroup analysis of the CAMMS223 trial demonstrated a significant improvement in EDSS scores even in patients without pre-treatment clinical disease activity (EDSS improvement by 0.31 points, *P* < 0.005) indicating potential neuroprotective effects of alemtuzumab [15]. In vitro experiments with peripheral blood mononuclear cells of alemtuzumab treated patients produced increased amounts of neurotrophins and were able to prolong survival of murine neurons and oligodendrocytes [15]. However, immune cells producing neurotrophins have not been detected in the CNS of alemtuzumab treated patients so far. Moreover, the short half-lives and the inability to pass the blood-brain-barrier (BBB) of neurotrophins argue against an effect of peripherally detected neurotrophic factors in the CNS [49]. Combining longitudinal clinical (SAD, SRD), MRI (brain atrophy, structural integrity) and experimental (neurotrophins and markers for neurodegeneration in the CSF, impact on neuronal networks in vitro) our study intends to evaluate potential neuroprotective properties of alemtuzumab.

A “two-hit model” of lymphopenia-associated autoimmunity is currently the prevailing hypothesis explaining the occurrence of secondary autoimmunity post alemtuzumab treatment [50]. The “first hit” is supposed to be triggered by the lymphopenia enabling self-antigen responsive T cells to proliferate after escaping depletion [51, 52]. Apart from genetic or environmental factors, increased IL-21 levels are proposed as potential “second hit” by increasing T cell cycling consequently enhancing the stochastic probability of self-antigen encounter [53]. Moreover, IL-21 is associated with the induction of T_h_ 17 cells [54], B cell differentiation, antibody production [55] and inhibition of Treg function [56, 57] potentially promoting secondary autoimmune disorders. Therefore, IL-21 has been suggested as a potential biomarker for the risk of secondary autoimmunity by Jones and colleagues [53], however verification in a large prospective cohort is still missing [58]. Moreover, the original ELISA kit used in the study by Jones and colleagues is not commercially available anymore and currently purchasable options have shown reduced predictive value [58] potentially limiting the conclusions drawn from the IL-21 ELISA in our study. However, with the combination of an in-depth phenotyping and a detailed surveillance of secondary autoimmune responses our study might be able to identify new risk markers.

Besides risk markers, it is a big unmet need to identify reliable markers for treatment response to allow for better therapy guidance. A differential lymphocyte reconstitution, which has been proposed as a potential marker, was not confirmed in subsequent studies [6, 59]. The aforementioned analysis of T cell diversity might provide a new opportunity in this matter since a recent report demonstrated differences in the rate and quality of TCR diversity restoration [7].

We are fully aware that the defined number of patients limits the possibility of definite conclusions; however this is not the focus of our study. Our study is meant as a “nested discovery cohort” for questions related to measuring immune reprogramming, potential neuroprotective mechanisms as well as lymphopenia-associated secondary autoimmunity. The results will pave the way for further investigations with larger cohorts evaluating and validating our findings.

### Trial status

Recruitment on-going.

## Additional file

Additional file 1: Assessment Schedule. (DOCX 42 kb)

## Abbreviations

anti-TPO: anti-thyroid peroxidase antibody
anti-TSHR: anti-thyroid stimulating hormone receptor antibodies
ARR: annual relapse reduction
BBB: blood-brain-barrier
BDNF: brain-derived neurotrophic factor
CNTF: ciliary neurotrophic factor
CS: complexity score
CSF: cerebrospinal fluid
CSFE: carboxyfluorescein succinimdyl ester
CTCAE: common terminology criteria for adverse events v4.0
DIC: disseminated intravascular coagulation
DMT: disease-modifying therapy
EBV: epstein-barr virus
EDSS: expanded disability status scale
EMA: european medicines agency
EQ-5D: euroqol
FAMS: functional assessment of multiple sclerosis
FDA: U.S. Food and Drug Administration
FLAIR: fluid attenuated inversion recovery
FSMC: fatique scale for motor and cognitive functions
GFAP: glial fibrillary acidic protein
HCMV: human cytomegaly virus
HIV: human immunodeficiency virus
HPV: human papilloma virus
HRQoL: health-related quality of life
huCD52 mice: transgenic human cd52 mice model
IAR: infusion associated reactions
ICF: informed consent form
IFNb-1a: interferon beta-1a
ITP: immune thrombocytopenic purpura
ITT: intention to treat
IUD: intrauterine device
LLN: lower limit of normal
MOA: mechanism of action
MRI: magnetic resonance imaging
MS: multiple sclerosis
MSFC: multiple sclerosis functional composite
NAA: N-acetylaspartate
NCI: National Cancer Institute
NEDA: no evidence of disease activity
NGF: nerve growth factor
NSE: neuron-specific enolase
NT-3: neurotrophin-3
NT-4: neurotrophin-4
OCT: optical coherence tomography
PAP: papanicolaou
PBMC: peripheral blood mononuclear cells
PP-analysis: per protocol analysis
RRMS: relapsing-remitting multiple sclerosis
SAD: sustained accumulation of disability
SC: subcutaneous
SF-36: short form-36
SmPC: summary of product characteristics
SPMS: secondary progressive ms
SRD: sustained reduction in disability
STIKO: ständige impfkommission
TCR: T cell receptor
Th: T helper cell
Treg: regulatory T cells
US: United States
VZV: varizella zoster virus

## References

[1] Crino L, Scagliotti G, Marangolo M, Figoli F, Clerici M, De Marinis F (1997). Cisplatin-gemcitabine combination in advanced non-small-cell lung cancer: a phase II study. J Clin Oncol.

[2] Martin R, McFarland HF, McFarlin DE (1992). Immunological aspects of demyelinating diseases. Annu Rev Immunol.

[3] Moreau T, Coles A, Wing M, Thorpe J, Miller D, Moseley I (1996). CAMPATH-IH in multiple sclerosis. Mult Scler.

[4] Hu Y, Turner MJ, Shields J, Gale MS, Hutto E, Roberts BL (2009). Investigation of the mechanism of action of Alemtuzumab in a human CD52 transgenic mouse model. Immunology.

[5] Rao SP, Sancho J, Campos-Rivera J, Boutin PM, Severy PB, Weeden T (2012). Human peripheral blood mononuclear cells exhibit heterogeneous CD52 expression levels and show differential sensitivity to Alemtuzumab mediated cytolysis. PLoS One.

[6] Cossburn MD, Harding K, Ingram G, El-Shanawany T, Heaps A, Pickersgill TP (2013). Clinical relevance of differential lymphocyte recovery after alemtuzumab therapy for multiple sclerosis. Neurology.

[7] Jones JL, Thompson SA, Loh P, Davies JL, Tuohy OC, Curry AJ (2013). Human autoimmunity after lymphocyte depletion is caused by homeostatic T-cell proliferation. Proc Natl Acad Sci U S A.

[8] Cox AL, Thompson SA, Jones J, Robertson VH, Hale G, Waldmann H (2005). Lymphocyte homeostasis following therapeutic lymphocyte depletion in multiple sclerosis. Eur J Immunol.

[9] Ruck T, Bittner S, Wiendl H, Meuth SG (2015). Alemtuzumab in multiple sclerosis: mechanism of action and beyond. Int J Mol Sci.

[10] Coles AJ, Wing MG, Molyneux P, Paolillo A, Davie CM, Hale G (1999). Monoclonal antibody treatment exposes three mechanisms underlying the clinical course of multiple sclerosis. Ann Neurol.

[11] Moreau T, Thorpe J, Miller D, Moseley I, Hale G, Waldmann H (1994). Preliminary evidence from magnetic resonance imaging for reduction in disease activity after lymphocyte depletion in multiple sclerosis. Lancet.

[12] Coles A, Deans J, Compston A (2004). Campath-1H treatment of multiple sclerosis: lessons from the bedside for the bench. Clin Neurol Neurosurg.

[13] Hirst CL, Pace A, Pickersgill TP, Jones R, McLean BN, Zajicek JP (2008). Campath 1-H treatment in patients with aggressive relapsing remitting multiple sclerosis. J Neurol.

[14] Investigators CT, Coles AJ, Compston DA, Selmaj KW, Lake SL, Moran S (2008). Alemtuzumab vs. Interferon beta-1a in early multiple sclerosis. N Engl J Med.

[15] Jones JL, Anderson JM, Phuah CL, Fox EJ, Selmaj K, Margolin D (2010). Improvement in disability after Alemtuzumab treatment of multiple sclerosis is associated with neuroprotective autoimmunity. Brain : a journal of neurology.

[16] Cohen JA, Coles AJ, Arnold DL, Confavreux C, Fox EJ, Hartung H-P (2012). Alemtuzumab versus interferon beta 1a as first-line treatment for patients with relapsing-remitting multiple sclerosis: a randomised controlled phase 3 trial. Lancet.

[17] Coles AJ, Twyman CL, Arnold DL, Cohen JA, Confavreux C, Fox EJ (2012). Alemtuzumab for patients with relapsing multiple sclerosis after disease-modifying therapy: a randomised controlled phase 3 trial. Lancet.

[18] Compston DAS GG, Arnold DL, Fox EJ, Hartung H-P, Havrdova E (2014). Durable effect of Alemtuzumab on clinical outcomes in patients with relapsing-remitting multiple sclerosis who relapsed on prior therapy: 4-year follow-up of care-ms i.

[19] Havrdova E,GG, Arnold DL, Coles AJ, Fox EJ, Hartung H-P (2014). Durable effect of Alemtuzumab on clinical outcomes in patients with relapsing-remitting multiple sclerosis who relapsed on prior therapy: 4-year follow-up of care-ms ii.

[20] LaGanke C, Hughes B, Berkovich R, Cohen J, Giovannoni G, Kasten L et al. Durable Effect of Alemtuzumab on Disability Improvement in Patients With Relapsing-Remitting Multiple Sclerosis Who Relapsed on a Prior Therapy (P3.261). Neurology. 2015;84(14 Supplement). Presented at the AAN Meeting 2015 in Washington.

[21] Arnold D, Traboulsee A, Coles A, Cohen J, Fox E, Hartung H-P et al. Durable Effect of Alemtuzumab on MRI Activity in Treatment-Naive Active Relapsing-Remitting Multiple Sclerosis Patients: 4-Year Follow-up of CARE-MS I (P7.246). Neurology. 2015;84(14 Supplement). Presented at the AAN Meeting 2015 in Washington.

[22] Traboulsee A, Coles A, Cohen J, Compston DAS, Fox E, Hartung H-P et al. Durable Effect of Alemtuzumab on MRI Outcomes in Patients With Relapsing-Remitting Multiple Sclerosis Who Relapsed on Prior Therapy: 4-Year Follow-up of CARE-MS II (P7.249). Neurology. 2015;84(14 Supplement). Presented at the AAN Meeting 2015 in Washington.

[23] Hartung H-P, Giovannoni G, Arnold D, Coles A, Fox E, Havrdova E et al. Improvement in Clinical Outcomes in Treatment-Naive Relapsing-Remitting Multiple Sclerosis Patients Who Switched From Subcutaneous Interferon Beta-1a to Alemtuzumab (P7.270). Neurology. 2015;84(14 Supplement). Presented at the AAN Meeting 2015 in Washington.

[24] Fox E, Giovannoni G, Arnold D, Coles A, Hartung H-P, Havrdova E et al. Improvement in Clinical Outcomes Following Switch From Subcutaneous Interferon Beta-1a to Alemtuzumab: CARE-MS II Extension Study (P7.278). Neurology. 2015;84(14 Supplement). Presented at the AAN Meeting 2015 in Washington.

[25] Barkhof F, Pelletier D, Coles A, Cohen J, Compston DAS, Fox E et al. Switching to Alemtuzumab From Subcutaneous Interferon Beta-1a After CARE-MS I Further Improved MRI Outcomes in Patients With Relapsing-Remitting Multiple Sclerosis (P7.261). Neurology. 2015;84(14 Supplement). Presented at the AAN Meeting 2015 in Washington.

[26] Pelletier D, Barkhof F, Coles A, Cohen J, Compston A, Fox E et al. Switching to Alemtuzumab From Subcutaneous Interferon Beta-1a After CARE-MS II Further Improved MRI Outcomes in Patients With Relapsing-Remitting Multiple Sclerosis (P7.248). Neurology. 2015;84(14 Supplement). Presented at the AAN Meeting 2015 in Washington.

[27] Cohen J, Arnold D, Coles A, Fox E, Hartung H-P, Havrdova E et al. Slowing of Brain Volume Loss in Patients With Relapsing-Remitting Multiple Sclerosis After Switching From Subcutaneous Interferon Beta-1a to Alemtuzumab (P7.264). Neurology. 2015;84(14 Supplement). Presented at the AAN Meeting 2015 in Washington.

[28] Tuohy O, Costelloe L, Hill-Cawthorne G, Bjornson I, Harding K, Robertson N (2015). Alemtuzumab treatment of multiple sclerosis: long-term safety and efficacy. J Neurol Neurosurg Psychiatry.

[29] Thompson SA, Jones JL, Cox AL, Compston DA, Coles AJ (2010). B-cell reconstitution and BAFF after Alemtuzumab (campath-1H) treatment of multiple sclerosis. J Clin Immunol.

[30] Feger U, Tolosa E, Huang YH, Waschbisch A, Biedermann T, Melms A (2007). HLA-G expression defines a novel regulatory T-cell subset present in human peripheral blood and sites of inflammation. Blood.

[31] Schwab N, Bien CG, Waschbisch A, Becker A, Vince GH, Dornmair K (2009). CD8+ T-cell clones dominate brain infiltrates in Rasmussen encephalitis and persist in the periphery. Brain : a journal of neurology.

[32] Arden B, Clark S, Kabelitz D, Mak TW (1995). Human T-cell receptor variable gene segment families. Immunogenetics. Immunogenetics.

[33] Pannetier C, Even J, Kourilsky P (1995). T-cell repertoire diversity and clonal expansions in normal and clinical samples. Immunol Today.

[34] Gobel K, Pankratz S, Schneider-Hohendorf T, Bittner S, Schuhmann MK, Langer HF (2011). Blockade of the kinin receptor B1 protects from autoimmune CNS disease by reducing leukocyte trafficking. J Autoimmun.

[35] Huang YH, Zozulya AL, Weidenfeller C, Metz I, Buck D, Toyka KV (2009). Specific central nervous system recruitment of HLA-G(+) regulatory T cells in multiple sclerosis. Ann Neurol.

[36] Herrmann AM, Gobel K, Simon OJ, Melzer N, Schuhmann MK, Stenner MP (2010). Glatiramer acetate attenuates pro-inflammatory T cell responses but does not directly protect neurons from inflammatory cell death. Am J Pathol.

[37] Meuth SG, Herrmann AM, Simon OJ, Siffrin V,NM, Bittner S (2009). Cytotoxic CD8+ T cell-neuron interactions: perforin-dependent electrical silencing precedes but is not causally linked to neuronal cell death. J Neurosci.

[38] Illes S, Fleischer W, Siebler M, Hartung HP, Dihne M (2007). Development and pharmacological modulation of embryonic stem cell-derived neuronal network activity. Exp Neurol.

[39] Jung Henson L, Arnold D, Cohen J, Coles A, Fox E, Hartung H-P et al. Incidence of Infection Decreases Over Time in Alemtuzumab-Treated Patients With Relapsing-Remitting Multiple Sclerosis: 4-Year Follow-up of the CARE-MS Studies (P7.265). Neurology. 2015;84(14 Supplement). Presented at the AAN Meeting 2015 in Washington.

[40] Coles AJ, Cox A, Le Page E, Jones J, Trip SA, Deans J (2006). The window of therapeutic opportunity in multiple sclerosis: evidence from monoclonal antibody therapy. J Neurol.

[41] Buggins AGS, Mufti GJ, Salisbury J, Codd J, Westwood N, Arno M (2002). Peripheral blood but not tissue dendritic cells express CD52 and are depleted by treatment with Alemtuzumab. Blood.

[42] Zhang X, Tao Y, Marcus K, Chopra M, Troiani L, Choudhary N (2012). Alemtuzumab (anti-human CD52 mAb) induces expansion of treg and Th2-cells and decreases frequencies of Th1- and Th17-cells in treated patients with relapsing remitting multiple sclerosis.

[43] Junker A, Ivanidze J, Malotka J, Eiglmeier I, Lassmann H, Wekerle H (2007). Multiple sclerosis: T-cell receptor expression in distinct brain regions. Brain.

[44] Batoulis H, Addicks K, Kuerten S (2010). Emerging concepts in autoimmune encephalomyelitis beyond the CD4/T(H)1 paradigm. Annals of anatomy. Anat An : official organ of the Anatomische Gesellschaft.

[45] Compston A, Coles A (2008). Multiple sclerosis. Lancet.

[46] Prat A, Biernacki K, Lavoie JF, Poirier J, Duquette P, Antel JP. Migration of multiple sclerosis lymphocytes through brain endothelium. Archives of Neurology. 2002;59(3):391–7. doi:10.1001/Archneur.59.3.391. 10.1001/archneur.59.3.391 11890842

[47] Havari E, Turner M, Dodge J, Treleaven C, Shihabuddin L, Roberts B (2014). Anti-murine CD52 antibody treatment does Not adversely affect the migratory ability of immune cells (P1.222). Neurology.

[48] J-i M, Yoshio T, Suzuki K, Kitagawa S, Iwamoto M, Kamimura T (1999). Characterization of the 4C8 antigen involved in transendothelial migration of CD26(hi) T cells after tight adhesion to human umbilical vein endothelial cell monolayers. J Exp Med.

[49] Thorne R, Frey W (2001). Delivery of Neurotrophic factors to the central nervous system. Clin Pharmacokinet.

[50] Krupica T, Fry TJ, Mackall CL (2006). Autoimmunity during lymphopenia: a two-hit model. Clin Immunol.

[51] Zandman-Goddard G, Shoenfeld Y (2002). HIV and autoimmunity. Autoimmun Rev.

[52] Powrie F, Leach MW, Mauze S, Caddle LB, Coffman RL (1993). Phenotypically distinct subsets of CD4+ T cells induce or protect from chronic intestinal inflammation in C. B-17 scid mice. Int Immunol.

[53] Jones JL, Phuah CL, Cox AL, Thompson SA, Ban M, Shawcross J (2009). IL-21 drives secondary autoimmunity in patients with multiple sclerosis, following therapeutic lymphocyte depletion with Alemtuzumab (campath-1H). J Clin Invest.

[54] Yang L, Anderson DE, Baecher-Allan C, Hastings WD, Bettelli E, Oukka M (2008). IL-21 and TGF-beta are required for differentiation of human T(H)17 cells. Nature.

[55] Ettinger R, Sims GP, Fairhurst AM, Robbins R, da Silva YS, Spolski R (2005). IL-21 induces differentiation of human naive and memory B cells into antibody-secreting plasma cells. J Immunol.

[56] Clough LE, Wang CJ, Schmidt EM, Booth G, Hou TZ, Ryan GA (2008). Release from regulatory T cell-mediated suppression during the onset of tissue-specific autoimmunity is associated with elevated IL-21. J Immunol.

[57] Peluso I, Fantini MC, Fina D, Caruso R, Boirivant M, MacDonald TT (2007). IL-21 counteracts the regulatory T cell-mediated suppression of human CD4+ T lymphocytes. J Immunol.

[58] Azzopardi L, Thompson SA, Harding KE, Cossburn M, Robertson N, Compston A (2014). Predicting autoimmunity after Alemtuzumab treatment of multiple sclerosis. J Neurol Neurosurg Psychiatry.

[59] Kousin-Ezewu O, Azzopardi L, Parker RA, Tuohy O, Compston A, Coles A (2014). Accelerated lymphocyte recovery after Alemtuzumab does not predict multiple sclerosis activity. American Academy of Neurology.

