# Supplementary material for: ALAIN01—Alemtuzumab in autoimmune inflammatory neurodegeneration: mechanisms of action and neuroprotective potential
Source: BMC Neurol. 2016 Mar 10;16:34. doi: 10.1186/s12883-016-0556-9 (PMC4785638; doi:10.1186/s12883-016-0556-9)
Supplement: Additional file 1: — Assessment Schedule. (DOCX 42 kb) [file 12883_2016_556_MOESM1_ESM.docx]

## Assessment Schedule, Flow Chart

|  |  | Scree-ning | Base-line | Month | | | | | | | | | | | | Unscheduled | |
| --- | --- | --- | --- | --- | --- | --- | --- | --- | --- | --- | --- | --- | --- | --- | --- | --- | --- |
|  |  |  |  | 3 | 6 | 9 | 12 | 15 | 18 | 21 | 24 | 27 | 30 | 33 | 36 | Re-lapse | Dis-cont. |
|  | Informed consent | x |  |  |  |  |  |  |  |  |  |  |  |  |  |  |  |
|  | Demographic data | X |  |  |  |  |  |  |  |  |  |  |  |  |  |  |  |
| **Safety assessments** | Medical history, concomitant diseases | x |  |  |  |  |  |  |  |  |  |  |  |  |  |  |  |
|  | Concomitant medication | x | X | x^1^ | x | x^1^ | x | x^1^ | x | x^1^ | x | x^1^ | x | x^1^ | x | X | x |
|  | Adverse Events | x | x | x^1^ | x | x^1^ | x | x^1^ | x | x^1^ | x | x^1^ | x | x^1^ | x | X | x |
|  | Vital signs | x |  | x^1^ | x | x^1^ | x | x^1^ | x | x^1^ | x | x^1^ | x | x^1^ | x | X | x |
|  | Physical examination | x |  | x^1^ | x | x^1^ | x | x^1^ | x | x^1^ | x | x^1^ | x | x^1^ | x | X | x |
|  | Blood pregnancy test | x |  |  |  |  |  |  |  |  |  |  |  |  |  |  |  |
|  | Blood or urine dipstick pregnancy test | Prior to each cycle of alemtuzumab and each MRI.  Blood test: within 3 days prior to each date. Urine test: on the respective date. | | | | | | | | | | | | | | | |
|  | Clinical chemistry^2^ | x |  | x^1^ | x | x^1^ | x | x^1^ | x | x^1^ | x | x^1^ | x | x^1^ | x |  | x |
|  | Creatinine | x |  | At least monthly | | | | | | | | | | | |  | x |
|  | Hematology^3^ | x |  | At least monthly | | | | | | | | | | | |  | x |
|  | Urinalysis^4^ | X |  | At least monthly | | | | | | | | | | | |  | X |
|  | Thyroid function^5^ | x |  | At least quarterly | | | | | | | | | | | |  | x |
|  | Basic lymphocyte phenotyping | x |  | x^1^ | x | x^1^ | x | x^1^ | x | x^1^ | x | x^1^ | x | x^1^ | x |  | x |
|  | Markers of autoimmunity^6^ |  | x |  | x |  | x |  | x |  | x |  | x |  | x |  | x |
|  | Screening for HIV, hepatitis B and C | x |  |  |  |  | x |  |  |  |  |  |  |  |  |  |  |
|  | Screening for HCMV, EBV, VZV | x |  |  |  |  | x |  |  |  |  |  |  |  |  |  |  |
|  | Cervical smear (cytology, HPV screening)) | X |  |  |  |  | x |  |  |  | x |  |  |  | x |  |  |
|  | Test for tuberculosis^7^ | X |  |  |  |  |  |  |  |  |  |  |  |  |  |  |  |
| **Efficacy assessments** | Blood sampling for efficacy assessments^8^ |  | x |  | x^1^ |  | x |  | x^1^ |  | x |  | x^1^ |  | x |  | x |
|  | CSF sampling^9^ |  | x^1^ |  |  |  | x^1^ |  |  |  | x^1^ |  |  |  | x^1^ |  | x^1^ |
|  | IL-21 expression |  | x |  |  |  | x |  |  |  | x |  |  |  | x |  | x |
|  | Expanded Disability Status Scale (EDSS) | x | x | x^1^ | x^1^ | x^1^ | x | x^1^ | x^1^ | x^1^ | x | x^1^ | x^1^ | x^1^ | x | X | x |
|  | Multiple Sclerosis Functional Composite (MSFC) |  | x | x^1^ | x^1^ | x^1^ | x | x^1^ | x^1^ | x^1^ | x | x^1^ | x^1^ | x^1^ | x |  | x |
|  | Quality of life (FAMS, EQ-5D, SF-36) |  | x | X^1^ | X^1^ | X^1^ | X | X^1^ | X^1^ | X^1^ | X | X^1^ | X^1^ | X^1^ | X |  | x |
|  | Fatigue Score (FSMC) |  | x | x^1^ | x^1^ | x^1^ | x | x^1^ | x^1^ | x^1^ | x | x^1^ | x^1^ | x^1^ | x |  | x |
|  | Optical Coherence tomography (OCT) |  | x |  | x^1^ |  | x |  | x^1^ |  | x |  | x^1^ |  | x |  | x |
|  | Evoked potentials (VEP, SEP, MEP) |  | x |  | x^1^ |  | x |  | x^1^ |  | x |  | x^1^ |  | x |  | x |
|  | MRI | x^10^ | x^11^ |  | x^1^ |  | x |  | x^1^ |  | x |  | x^1^ |  | x |  | x |
|  | Inclusion and exclusion criteria | x |  |  |  |  |  |  |  |  |  |  |  |  |  |  |  |
|  | Criteria for treatment deferral |  |  |  |  |  | X |  |  |  |  |  |  |  |  |  |  |
|  | Administration of trial medication (premedication: methylprednisolone) |  | x |  |  |  | x |  |  |  |  |  |  |  |  |  |  |
|  | Start of anti-infective prophylaxis |  | x |  |  |  | x |  |  |  |  |  |  |  |  |  |  |
|  | Recent symptoms prior to relapse |  |  |  |  |  |  |  |  |  |  |  |  |  |  | x |  |
|  | Supportive laboratory testing in case of suspected relapse^12^ |  |  |  |  |  |  |  |  |  |  |  |  |  |  | X |  |
|  | Determination whether patient had relapse |  |  |  |  |  |  |  |  |  |  |  |  |  |  | x |  |
|  | Diary delivery and/or collection | x | x | x^1^ | x | x^1^ | x | x^1^ | x | x^1^ | x | x^1^ | x | x^1^ | x | x | x |
|  | Patient alert card delivery/renewal |  | x |  |  |  | x |  |  |  |  |  |  |  | x |  | x |

^1^ Not mandatory

^2^ Clinical chemistry: sodium, potassium, ALT, AST, glucose

^3^ Hematology CBC with platelet count; differential WBC, Hgb, Hct,

^4^ Urinalysis: colour, appearance, specific gravity, pH, protein, glucose, ketones, bilirubin, hemoglobin

^5^ Thyroid function: TSH, free T3, free T4, anti-TSHR, anti-TPO antibodies

^6^ ANA, cANCA, pANCA, anti-dsDNA, anti-TSHR, anti-TPO, RF, anti-CCP, anti-GBM, anti-platelet antibodies

^7^ 2 interferon-gamma release assays (QuantiFERON®-TB Gold In-Tube test, T-SPOT®.TB test)

^8^ Blood efficacy assessments: Identification and quantification of immune cell populations, functional characterization of T cell subsets, concentration of neurotrophic factors

^9^ CSF-lymphocyte phenotyping: Functional characterization of T cell subsets, concentration of neurotrophic factors, evaluation of markers for neurodegeneration, effects of supernatant samples on neuroactivity

^10^ If previous MRI is not available to assess inclusion criteria

^11^ Not mandatory if already performed within 3 month prior initiation of Lemtrada®, provided that the MRI fulfils the study specific requirements and the patient is clinically stable (no new symptoms for at least 3 months and no therapy for a relapse) within this time period

^12^ CRP, leukocytes, urine testing for infections
